# Supplementary material for: Surveillance of CKD epidemiology in the US – a joint analysis of NHANES and KEEP
Source: Sci Rep. 2018 Oct 26;8:15900. doi: 10.1038/s41598-018-34233-w (PMC6203800; doi:10.1038/s41598-018-34233-w)
Supplement: Supplementary file 1 — Supplementary Tables [file 41598_2018_34233_MOESM1_ESM.pdf]

Surveillance of CKD epidemiology in the US – a joint analysis of NHANES and KEEP

Myers OB[1], Pankratz VS[1], Norris KC[2], Vassalotti JA[3], Unruh M[1], Argyropoulos C[1,*]

[1]Internal Medicine, University of New Mexico, Albuquerque, NM 87131

[2]David Geffen School of Medicine, UCLA, Los Angeles, CA 90024

[3]National Kidney Foundation Inc., New York, New York

*Correspondence to : cargyropoulos@salud.unm.edu

Supplementary Tables

Supplemental Table 1. Prevalence of CKD Stage 3 and Stage 4-5 by sex and age from NHANES and KEEP (2001-2012) without and with inverse probability weighting (% and 95% CI). NHANES estimates accounted for sampling design. KEEP WTD-GEE estimates accounted for participant self-selection and clustering within regional affiliates. CKD – chronic kidney disease; KEEP – Kidney Early Evaluation Program; NHANES - National Health and Nutrition Examination Survey

		Stage 3			Stage 4-5		
Sex	Age	NHANES	KEEP	KEEP WTD-GEE	NHANES	KEEP	KEEP WTD-GEE
Female	20-39	0.06 (0.02-0.18)	0.84 (0.70-1.00)	0.55 (0.34-0.89)	0.07 (0.02-0.20)	0.12 (0.08-0.19)	0.01 (0.00-0.02)
	40-59	2.52 (1.95-3.25)	5.44 (5.21-5.67)	4.46 (3.80-5.22)	0.12 (0.07-0.21)	0.34 (0.29-0.41)	0.17 (0.11-0.25)
	60-69	11.49 (9.68-13.59)	17.06 (16.53-17.61)	15.19 (13.54-17.01)	0.52 (0.33-0.82)	0.89 (0.77-1.04)	0.41 (0.27-0.63)
	70+	36.25 (34.23-38.32)	39.50 (38.73-40.28)	39.24 (37.17-41.34)	3.74 (3.05-4.58)	3.02 (2.77-3.31)	3.42 (2.66-4.40)
Male	20-39	0.24 (0.12-0.49)	1.64 (1.36-1.97)	0.88 (0.63-1.23)	0.01 (0.00-0.10)	0.29 (0.19-0.45)	0.07 (0.03-0.14)
	40-59	1.74 (1.30-2.32)	5.40 (5.08-5.75)	4.01 (3.25-4.96)	0.04 (0.02-0.11)	0.53 (0.43-0.65)	0.13 (0.07-0.21)
	60-69	9.84 (8.55-11.31)	16.09 (15.34-16.87)	13.67 (12.29-15.17)	0.65 (0.34-1.22)	1.11 (0.91-1.35)	0.74 (0.51-1.06)
	70+	31.74 (29.86-33.69)	36.24 (35.18-37.31)	35.66 (33.21-38.17)	2.72 (2.14-3.45)	3.56 (3.17-4.00)	3.41 (2.81-4.15)

Supplemental Table 2. Linear trend estimates for CKD Stage: NHANES and KEEP WTD-GEE. Coefficient estimates are for a two-year change. NHANES estimates account for sampling strata and clusters using two year sampling weights for mobile clinics, and are estimates for the domain that met selection criteria. KEEP estimates employed inverse probability weighting for self-selection and used GEE to account for affiliate sampling clusters.

	Coefficient (95% CI) for 2-yr Change			
Stage	NHANES	P-value	KEEP WTD-GEE	P-value
3	-0.00085 (-0.0210, 0.0193)	0.93	-0.0035 (-0.0591, 0.0522)	0.90
3-5	0.000192 (-0.0194, 0.0198)	0.98	-0.0038 (-0.0591, 0.0515)	0.89
4-5	0.0122 (-0.0376, 0.0620)	0.63	-0.0075 (-0.1303, 0.1153)	0.90
